# Long-range transcription factor binding sites clustered regions may mediate transcriptional regulation through phase-separation interactions in early human embryo

**DOI:** 10.1016/j.csbj.2024.09.017

**Published:** 2024-09-26

**Authors:** Mengge Tian, Xiaohan Tang, Zhangyi Ouyang, Yaru Li, Xuemei Bai, Bijia Chen, Shutong Yue, Pengzhen Hu, Xiaochen Bo, Chao Ren, Hebing Chen, Meisong Lu

**Affiliations:** a The First Affiliated Hospital of Harbin Medical University, Harbin 150001, China; b Academy of Military Medical Sciences, Beijing 100850, China; c Ruijin Hospital, Shanghai Jiao Tong University School of Medicine, Shanghai 200025, China; d School of Life Sciences, Northwestern Polytechnical University, Xi’an 710072, China

**Keywords:** Transcription factors binding sites clustered regions (TFCRs), Transcriptional regulation, Early embryo

## Abstract

In mammals, during the post-fertilization pre-implantation phase, the expression of cell type-specific genes is crucial for normal embryonic development, which is regulated by cis-regulatory elements (CREs). TFs control gene expression by interacting with CREs. Research shows that transcription factor binding sites (TFBSs) reflect the general characteristics of the regulatory genome. Here, we identified TFBSs from chromatin accessibility data in five stages of early human embryonic development, and quantified transcription factor binding sites-clustered regions (TFCRs) and their complexity (TC). Assigning TC values to TFCRs has made it possible to assess the functionality of these regulatory elements in a more quantitative way. Our findings reveal a robust correlation between TFCR complexity and gene expression starting from the 8Cell stage, which is when the zygotic genome is activated in humans. Furthermore, we have defined long-range TFCRs (LR-TFCRs) and conjecture that LR-TFCRs may regulate gene expression through phase-separation mechanisms during the early stages of human embryonic development.

## Graphical Abstract

**Figure:**
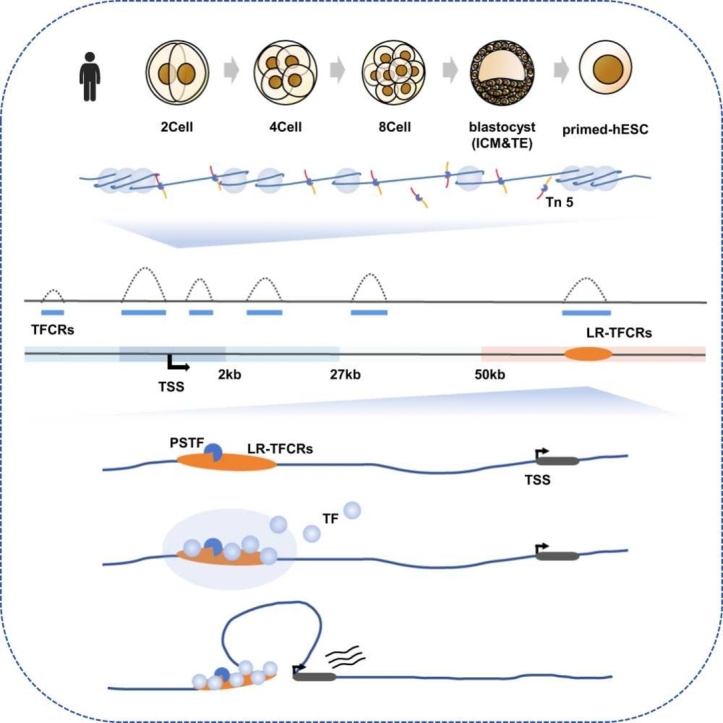

## 1 Introduction

Mammalian embryogenesis commences with the seminal event of sperm-oocyte fusion, leading to the genesis of a zygote. This nascent cell embarks on a transformative odyssey, undergoing fertilization, a series of mitotic divisions, and compaction to culminate in forming the compact morula. Subsequently, the process of polarization and cavitation transforms the morula into the hollow structure of the blastocyst. Throughout these stages, the embryo meticulously multiplies its cellular constituents while preserving its overall dimensions, a feat accomplished by utilizing of maternal mRNAs and proteins inherited from the oocyte [1]. Despite the embryo's initial reliance on maternally derived macromolecules, the zygotic genome remains quiescent, necessitating its eventual activation for the embryo to execute its developmental blueprint. The critical juncture of zygotic genome activation (ZGA) is heralded by the degradation of maternal components and the initiation of embryonic transcription, thereby endowing the embryo with the autonomy to direct its developmental trajectory [2]. ZGA is a pivotal and evolutionarily conserved milestone in the embryonic developmental program, albeit with temporal variations across species [3]. In murine models, ZGA transpires during the transition from the early to the late 2Cell stages of cellular division. Comparatively, in humans, this genomic awakening unfolds between the 4Cell and 8Cell embryonic stages [1]. The orchestration of ZGA is intricately linked to the synchronized upregulation of a cadre of genes and transcription factors, which collectively orchestrate the complex choreography of embryonic development [4], [5].

Recent studies underscore the pivotal role of epigenetic information in maintaining cellular identity and regulating gene expression [6]. Initially, during development, a state of overall hypomethylation is crucial for preserving the naive pluripotency of the embryo and for setting the stage for precise differentiation down the line [7]. A key regulatory event in pre-implantation embryogenesis is the reprogramming of histone modifications, which significantly influences how transcriptional regulators interact with chromatin [8], [9]. As embryos develop, studies utilizing low-input DNase I sequencing (liDNase-seq) have illustrated a progressive establishment of DNase I hypersensitive sites (DHSs), with a notable increase observed in 8Cell embryos [10], [11]. Concurrently, ATAC-seq (Analysis of Transposase-Accessible Chromatin Using Sequencing) studies have shed light on the dynamics of chromatin accessibility during early mouse embryo development [12], [13]. After fertilization in mammals, chromatin organization is subject to dramatic reprogramming. Evidence suggests that chromatin in the post-fertilized mouse embryo may initially be in a state of pronounced relaxation, which is followed by a progressive maturation of higher-order chromatin structures as the embryo develops [14], [15]. A correlative study examining the dynamics of chromosome structure during sperm and early human embryo development [16] has highlighted the essential regulatory function of CTCF proteins in shaping the topology associated with TAD (Topologically Associating Domains) structures in the early stages of embryogenesis. Intriguingly, this research indicates that while human topological domains are primarily formed gradually at the 8Cell stage, in mice, these domains begin to establish themselves from the 2Cell stage onward. The process of epigenetic reprogramming during early embryonic development is intricately regulated, involving a multitude of epigenetic marks. Furthermore, transitions in cell fate are orchestrated by various types of epigenetic reprogramming events [6].

The human genome is composed of more than 98 % non-coding DNA, which includes sequences with specific functional features such as cis-regulatory elements (CREs) and retrotransposons[17]. CREs constitute approximately 20–40 % of the non-coding region of the genome, which contains sites for enhancers, promoters, and chromatin interactions [18]. Studies have shown that proximal promoters are generally active across a broad spectrum of cell types, but the activation of distal enhancers is specific to each cell type [19]. Some CREs define cell type identity by establishing lineage-specific gene expression profiles [20]. With the development of sequencing technologies, ChIP-seq for histone modifications (e.g., H3K4me3 and H3K27ac) [21], chromatin-binding proteins (CTCF and ZNF143)[22], [23], or chromatin accessibility analysis (DNase-seq and ATAC-seq) [24], [25] have been used to annotate CREs in biological samples. Different types of CREs can be characterized by their epigenomic features, including DNA methylation, chromatin accessibility, histone modifications, and local chromatin structure. CREs are unevenly distributed across the genome, suggesting a different biological basis between clusters of CREs (COREs) and individual CREs. In 2019, Madani Tonekaboni et al. developed an unsupervised machine learning, CREAM, to automatically identify open chromatin data from COREs and demonstrated the enhanced value of COREs over super-enhancers in identifying master transcriptional regulators, highly expressed genes, and genes essential for defining cellular identity [26].

In recent years, studies have revealed that transcription factor binding is highly aggregated in the human genome [25], [27], [28], and this widespread presence of TFBSs suggests that they embody the general properties of regulatory genomes. A study developed a computational method [29] to calculate transcription factor binding sites clustering regions (TFCRs) and TFCRs complexity by identifying chromatin TFBSs in open chromatin data and proposed a model of transcriptional regulation determined by complexity that can be used for chromatin-accessible data. This approach allows for more precise identification of regulatory elements from chromatin-accessible data sources. It also enables a quantitative assessment of the roles and mechanisms of these regulatory elements. Previous studies[30], [31], [32] highlight the importance of TFCRs in gene regulation, particularly in the context of cancer and embryonic development. These provide new insights into how TFCRs function in different species and at different stages of development.

Therefore, we applied FIMO [33] to identify the TFBSs of 2Cell, 4Cell, 8Cell, ICM, and primed-hESC (GSE101571, ATAC-seq) [8] of early human embryos. We calculated TFCRs and the complexity of TFCRs by Gaussian fitting in expectation of exploring the dynamic changes of TFCR accessibility during the development of early human embryos.

### 1.1 Result 1 transcription factor binding sites clustered regions (TFCRs) of early human embryonic development

**Fig. 1:**
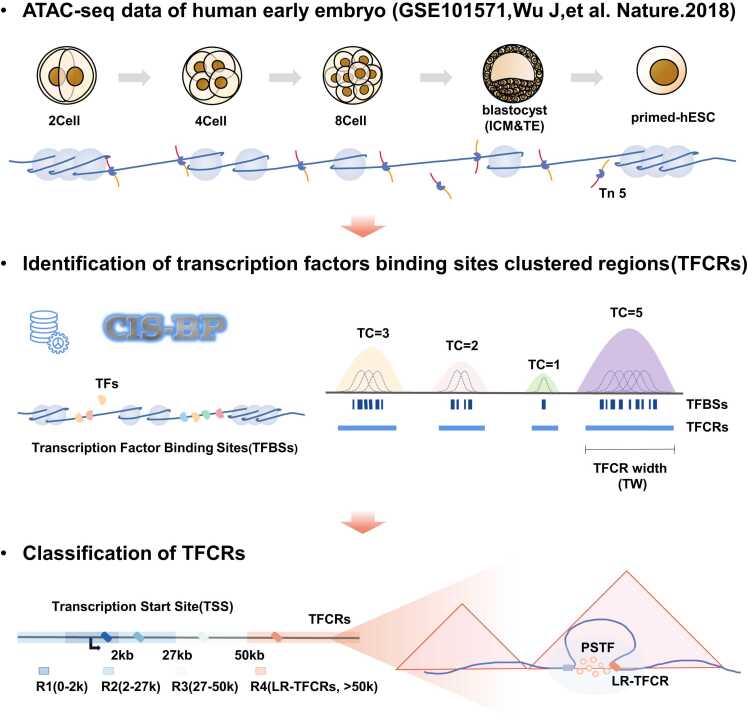
Schematic model of TFCR transcriptional regulation during early human embryonic development. Chromatin accessibility data (ATAC-seq) from GEO (GSE101571, Wu J et al., 2018) were collected for five stages, including 2Cell, 4Cell, 8Cell, ICM, and primed-hESC. TFBSs were scanned from these chromatin accessibility data using TF motifs from the FIMO and CIS-BP databases. TFCRs were identified and TFCR complexity (TC) was calculated, and unit base complexity, cw, was calculated based on the complexity versus the width of TFCRs. TFCRs were categorized according to the distance of TFCRs from the transcription start site (TSS) of genes. TFCRs with different distances from the genes’ TSS play different roles in early embryonic development, whereas long-range TFCRs (LR-TFCRs) located at the most distal end may play regulatory roles in gene transcription. LR-TFCRs may regulate the formation of three-dimensional chromatin structures through phase separation, thereby regulating gene expression.

We collected chromatin accessibility data[8] from the early human embryo (GSE101571), which covers five stages: 2Cell, 4Cell, 8Cell, ICM, and primed-hESC (Fig. 1, upper panel). TFBSs were scanned from these chromatin accessibility data using FIMO [33] with TFs' motifs from the CIS-BP database [34]. Next, we identified the TFCRs of human early embryos at each stage in a similar way as described in a previous study [29] and calculated the complexity of TFCRs (TC) (Fig. 1, middle panel). TC was determined by the quantity and proximity of the contributing TFBS. To explore the effect of TFCRs on the regulation of gene expression, we comprehensively evaluated TFCRs in terms of their complexity, width and by calculating the unit base complexity (cw). TFCR width (TW) is the distribution length of TFCR in the genome. The cw is the ratio of the complexity of TFCRs to the width of the TFCRs. Finally, we explored the effects of different regional TFCRs on transcriptional regulation through the classification of the distance between TFCRs and genes (Fig. 1, lower panel).

**Fig. 2:**
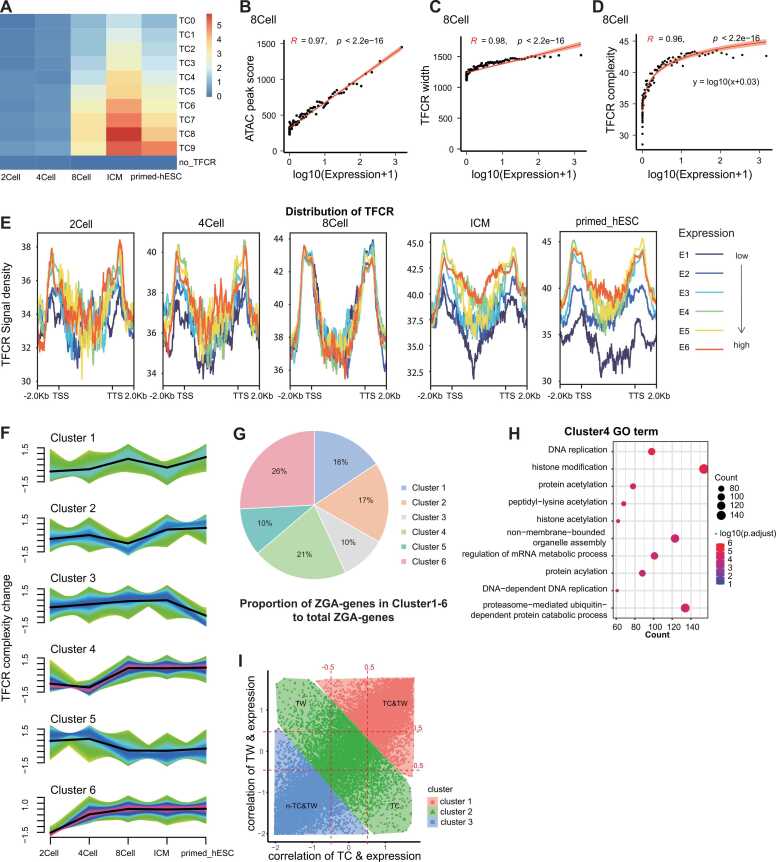
TFCRs regulatory model during early human embryonic development. **A.**Heatmap of gene expression levels of each TFCRs group (TC0-TC9,no_TFCR) at each stage of early embryonic development. **B.**Scatterplot of ATAC peak score versus gene expression level at 8Cell stage shows a strong correlation between them. Red section: fitting of TFCR width to gene expression, R represents the magnitude of correlation. **C.**Scatterplot of TFCR width versus gene expression level at 8Cell stage shows a strong correlation between them. Red section: fitting of TFCR width to gene expression, R represents the magnitude of correlation. **D.**Scatterplot of TFCR complexity versus gene expression levels at 8Cell stage shows a strong correlation between them. Red section: fitting of TFCR complexity with gene expression, R represents the correlation magnitude. **E.**The distribution of TFCRs corresponding to different genes was classified into six groups (E1-E6) according to gene expression from low to high. The vertical axis responses the TC values. TSS: gene transcription start site, TTS: gene transcription termination site. **F.**Plot of clustering using Mfuzz of changes in TFCR complexity with developmental progression. The horizontal axis shows the five stages of early embryonic development. The red part indicates that TFCR is more enriched under this trend. **G.**Pie chart of the proportion of ZGA genes contained in each clustering group to the total ZGA genes ratio. H.Gene ontology enrichment analysis of genes in Cluster4. I.Clustered scatterplot of correlation between TFCR complexity and gene expression versus correlation between TFCR width and gene expression using Kmeans. Taking |cor|> 0.5 divides into 4 groups (nTC&TW, TC, TC&TW, and TW).

Firstly, we compared the accessibility of TFCRs at the five stages, and found that it gradually increased (Fig. S1a). We found that the accessibility of TFCRs at ICM stages decreased because ICM is a part of the blastocyst stage. In early mammalian development, the totipotent fertilized egg undergoes the first cell fate determination, forming a blastocyst with an inner cell mass (ICM) and a trophoblast and blastocyst coelom structure[35]. Among them, ICM clusters go on to produce future pluripotent cells and eventually differentiate into various embryonic lineages. We observed a reduction in the accessibility of TFCRs during the ICM stage, which is consistent with the differentiation and allocation of blastocyst cells. We counted the number of ATAC peaks in the early embryo (Fig. S1b), and found that the number of peaks was relatively less in the ICM stage, which may be the significant factor affecting the number of TFCRs in the ICM stage. According to the co-localization relationship between TFCRs and gene promoter region, expressed genes are determined probably regulated by these TFCRs, and the matrix between gene expression and TFCRs complexity was constructed. We sorted the TFCRs in complexity ascending order, where the no_TFCRs group represented the absence of TFCRs in the genomic region, and TC0-TC9 represented the complexity from low to high. We categorized TFCRs into no_TFCR and TC0-TC9 according to TFCR complexity (TC)(Fig. 2A). By comparing the expression of genes in different TC classifications during the early embryonic development, we found that genes in groupings with higher TC possessed higher expression since the 8Cell stage. Therefore, we further explored the relationship between TFCR complexity and gene expression level during early human embryonic development. By plotting a scatterplot of chromatin accessibility (ATAC peak score) versus gene expression level (log10(Expression+1)) (Fig. 2B, Fig. S1c), the correlation between the ATAC peak score and gene expression level is significantly stronger since 8Cell stage than in the earlier stages. Recent studies have shown that genes with higher expression have larger numbers of CREs in their CRE landscapes [36]. TFCRs are transcription factor binding sites clustered regions identified by analysis of chromatin accessibility data, which play a key role in regulating gene expression. CREs are a class of highly conserved transcriptional regulatory regions that regulate gene expression during embryonic development. We suppose that TFCRs are functionally similar to CREs in that they are both involved in complex gene regulatory networks and may influence cell fate decisions and tissue development. The results showed that the width of TFCRs was strongly correlated with gene expression level since 8Cell stage (Fig. 2C, Fig. S1d). We also calculated the relationship between TC and gene expression level at various stages of early embryonic development, and we found a clear (y = log10(x + 0.03)) fit between them from 8Cell stage onwards. The result suggests a multifactorial influence on transcriptional regulation (Fig. 2D, Fig. S1e). The complexity of each TFCR was determined by the quantity and proximity of the contributing TFBS. The higher the TC, the more number and types of transcription factors that can be bound on TFCR. There is no significant change in the gene expression level when the TC is at a low level, and when the TC is increased, the expression level increases rapidly. The results revealed that the expression of genes regulated by TFCRs may undergo transcription factor enrichment, transcription surge, and saturation state. The formation of a saturation state may be caused by multiple factors regulating gene expression. Not only that, the distribution of TFCRs in each stage (Fig. 2E) illustrated that the early embryonic development of the initial stage (2Cell, 4Cell) did not show a significant change, but since the 8Cell stage onwards, TC associated with higher expression genes are higher. However, the TC of the E6 group (the highest expression group) was not the highest, which also proved that TFCRs were not the only condition to regulate gene expression.

Next, we clustered the TFCRs according to TC changes in five stages of early embryonic development into Cluster1–6 (Fig. 2F). We found that the pattern of change in TFCR complexity of Cluster4 and Cluster6 was similar to the transcription of ZGA genes. So we looked at the proportion of ZGA genes (Li et al. in preparation) in each cluster (Fig. 2G), with 47 % of ZGA genes presented in Cluster4 and Cluster6. Gene ontology (GO) enrichment analysis of ZGA genes under this classification revealed that Cluster4 was mainly enriched in DNA replication, histone modification, and regulation of the mRNA metabolic process (Fig. 2H). The TCs of TFCRs which regulate replication, transcription, and modification of functional genes involved in embryonic development gradually increases in order to regulate the genes to achieve the corresponding functions.

In order to explore the effects of TFCR complexity and width on gene expression, we used K-means to cluster genes into three groups according to the correlation between TFCR complexity, width, and associated genes (Fig. 2I). Based on the clustering, according to the correlation between gene expression level and the two features, we categorized the genes as being strongly correlated only with the complexity of the TFCRs (TC), strongly correlated only with the TFCR width (TW), correlated with both (TC & TW), and less correlated with both (n-TC&TW). The result of GO analysis showed that TC&TW was mainly enriched in the Wnt signaling pathway (Fig. S2a). A relevant study shows that the Wnt signaling pathway is one of the important signaling pathways in embryo and organ development, and is involved in the regulation of proliferation, differentiation, polarization, migration, and apoptosis of various cells in the organism [37].

Next, we calculated the density distribution of TFCR width (Fig. S2b), and the result shows differences in the distribution of TFCR width at different stages. To better explore the quantitative effect of the complexity of TFCRs, we attempted to calculate the unit base complexity of TFCRs (cw) to normalize eigenvalues of TFCRs to delve into their regulatory influence during early embryogenesis. We calculated the cw values at each stage of early embryonic development separately and explored the density distribution of cw (Fig. S2c). The result shows similarities in the distribution of cw at different stages. We sorted cw in descending order and then filtered out the top 200 TFCRs (top-200). We selected 200 TFCRs at random from the residual TFCRs pool to establish a control group, designated as "sampling-200." We undertook a rigorous statistical analysis to discern the genomic distribution patterns of TFCRs (Fig. S2d).

Interestingly, the result shows that TFCRs within the top-200 are predominantly situated within promoter regions, underscoring their strategic positioning in the genome. We further analyzed genes affiliated with the top-200 TFCRs throughout various stages (Fig. S2e). A starkly low concordance among the genes active at each stage, suggests a dynamic and stage-specific regulatory landscape. The result of the GO enrichment analysis shows that the pathway predominantly enriched, centered around embryonic development, including in utero embryonic development, pattern specification process, and the regulation of DNA-binding transcription factor activity (Fig. S2f).

It is not until the ZGA that a significant amount of TFCRs would be engaged in one or more functions to coordinate the activation of zygotic gene expression. Our focus has been redirected towards TFCRs ZGA, particularly during the 8Cell. We have identified and scrutinized 11 genes within the top-200 that emerge after ZGA. Notably, "SMAD2," a key mediator of the TGF-β signaling pathway, has come to the fore. Haipeng Fu et al. reported that the TGF-β signaling pathway could regulate the development of mesoendoderm not only by modulating the expression of transcription factors but also by steering cell fate decisions through the regulation of cellular glucose metabolism [38]. During development and regeneration, the Wnt and TGF-β signaling pathways not only function independently but also exhibit reciprocal regulation. For instance, in certain cell types, Wnt signaling can either activate or inhibit the expression of TGF-β target genes, while TGF-β can modulate β-catenin's activity through Smad proteins. In summary, the Wnt and TGF-β signaling pathways play a pivotal role in regulating cellular behavior and tissue development, with their interactions having profound implications for various biological processes and diseases. However, the precise mechanisms by which TFCRs govern these genes to influence embryonic progression remain to be elucidated through further experimental investigation.

These findings underscore the pivotal roles of these pathways in orchestrating embryonic development. From our observations, we conclude that TFCRs with higher cw values are conferred with strong transcriptional regulatory potency, thereby exerting a more pronounced influence on gene expression and cellular function. We found that TFCR complexity, as well as TFCR width, showed a strong correlation with gene expression since 8Cell stage, which was consistent with the drastic genetic changes that occur in 8Cell human embryos.

### 1.2 Result 2 patterns of TFCRs-mediated transcriptional regulation evolve and stabilize with embryonic developmental progression

**Fig. 3:**
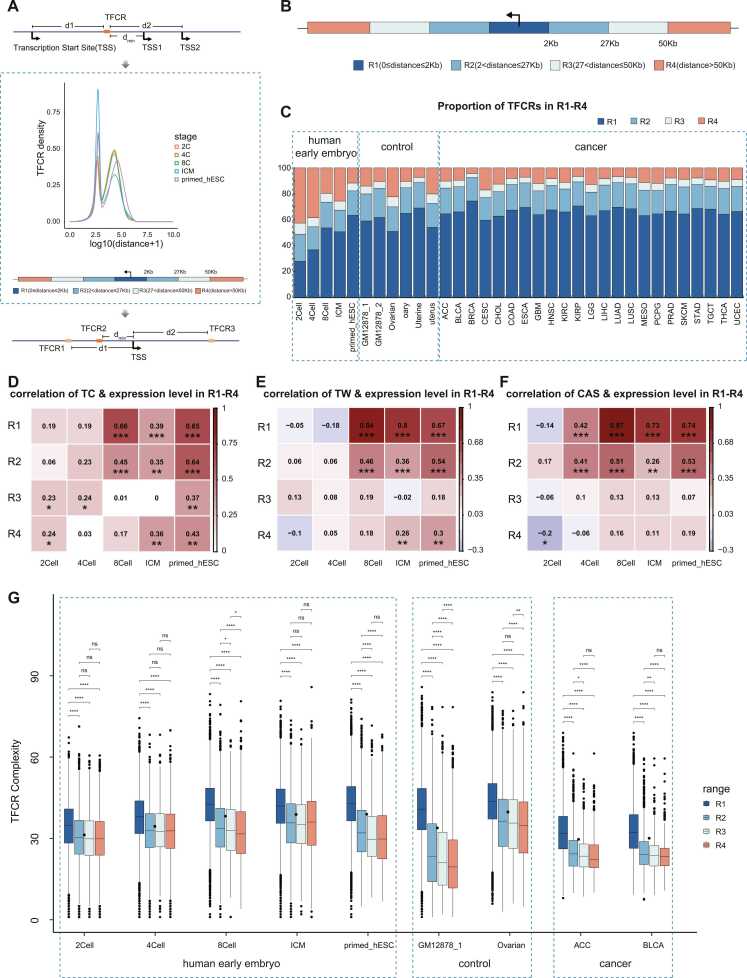
Classification and characterization of TFCR distances. **A.**Pattern diagram of distance classification methods for TFCRs. **B.**TFCRs region classification pattern illustration. R1 region indicates that the distance between TFCR and its associated genes is 0–2k (0 ≤ distance ≤ 2k), R2 region is 2–27k (2k < distance ≤ 27k), R3 region is 27–50k (27 < distance≤50k), and R4 region is 0–2k (distance>50k). **C.**Proportional distribution of TFCR in R1-R4 region across samples illustrates the gradual stabilisation of the proportion of TFCRs in each region. **D.**Heatmap showing the correlation between TFCR-related gene expression levels (log10(expression +1)) and TFCR complexity in R1-R4 for each sample of early embryos, ***p ≤ 0.001, **p ≤ 0.01, *p ≤ 0.05. **E.**Heatmap showing the correlation between the expression level (log10(Expression+1)) of TFCR-associated genes and TFCR width in R1-R4 for each sample of early embryos, ***p ≤ 0.001, **p ≤ 0.01, *p ≤ 0.05. **F.**Heatmap showing the correlation between the expression level(log10(Expression+1)) of TFCR-associated genes and CAS in R1-R4 for each sample of early embryos, ***p ≤ 0.001, **p ≤ 0.01, *p ≤ 0.05. **G.**Distribution of TFCR complexity by region across samples, ****p ≤ 0.0001, ***p ≤ 0.001, **p ≤ 0.01, *p ≤ 0.05, ns p > 0.05.

To further explore the regulatory mode of TFCRs, we classified accessible TFCRs based on the distance between TFCRs and the gene transcription start site (TSS). First, we took each TFCR as the center, compared the distance between the TFCR and the TSS of each gene, and screened the gene closest to TFCR for labeling (Fig. 3A). Then, we explored the density distribution of the TFCR and gene TSS distance in five stages (Fig. S3a), the plot showed two local maximum values and one local minimum value. The first local maximum is about 500 bp away from the gene, and the second local maximum is about 27k bp away from the gene. The local minimum is about 2k bp. Then we calculated 99 quantiles of the distance matrix density distribution between TFCRs and genes' TSS, and finally selected 2k bp, 27k bp, and 50k bp distances from genes to classify TFCRs, and divided TFCRs into four regions. To unify the standards for the role of regulation, we assume that each gene receives the regulatory action of the nearest TFCR, and we call this gene the associated gene of this TFCR. Then, we took each gene TSS as the center, compared the distance between each TFCR and the gene transcription start site, and screened the TFCRs closest to the gene TSS as the TFCR corresponding to the gene. We divided TFCRs into four regions based on this distance distribution (Fig. 3B), which are R1 (0 ≤distance≤2 kbp), R2 (2k<distance≤27 kbp), R3 (27k<distance≤50 kbp), and R4 (distance>50 kbp). In addition to data from various stages of human early embryonic development, we also collected and identified TFCRs from a total of 29 samples of other human cell lines and tissues, and performed R1-R4 classification (Fig. 3C). GM12878, uterine and ovarian samples provide a baseline for gene expression and regulation in mature tissues and help us understand regulatory changes during early embryonic development. And 23 cancer samples from the TCGA database (https://portal.gdc.cancer.gov/repository) were considered mature cancer tissue samples. During early embryonic development, the proportion of accessible TFCRs in the R1 region increases, R4 region decreases, and the proportion of accessible TFCRs in R2 and R3 was basically stable. The results showed that with the developmental process, the proportion of accessible TFCRs until the primed-hESC stage in the proportion of R1-R4 was similar to that of mature tissues.

Next, we explored the associated genes of TFCRs in the R1-R4 region and found that the highly expressed (H group, FPKM>1) genes also showed a trend of increasing in the R1 region and decreasing in the R4 region with developmental progression (Fig. S3b). The distribution of high and low expressed genes in R1-R4 at the primed-hESC stage was similar to mature tissues. In addition to TFCR complexity and TFCR width, chromatin accessibility score (CAS) [32] can well reflect the level of chromatin accessibility measured by ATAC-seq data corresponding to TFCR, so we explored each regional the TFCR from these three features. The CAS, complexity, and width of TFCRs of each sample also showed a high correlation with gene expression in the R1 region (Fig. 3D-F, Fig. S3c-g). Interestingly, in some samples, the TC in the R4 region still shows a strong correlation with gene expression. We suppose that TFCRs in the R4 region, although occupying a much smaller proportion, still play some role in gene expression. Compared with the other three regions, the complexity of TFCRs in the R1 region is higher (Fig. 3G). In summary, as embryonic development proceeds, the proportion of accessible TFCRs in each region gradually stabilize. We found that TFCRs in the R1 region correlated more strongly with gene expression level than TFCRs in other regions in terms of each eigenvalue. Moreover, the TC values of TFCRs in the R1 region were significantly higher than those in other regions. In summary, TFCRs in the R1 region play a crucial role in transcriptional regulation.

As early embryonic development progresses, each region of the TFCRs exhibits different characteristics. However, how TFCR accessibility continuously changes with development and gradually stabilizes, and how it plays a regulatory role in different regions, need to be further explored.

### 1.3 Result 3 proximal TFCRs and long-range TFCRs play an important role in transcriptional regulation

During embryonic development, the most obvious changes in the proportion of TFCRs are in the R1 and R4 regions, with an increasing proportion of TFCRs in the R1 region and a decreasing proportion of TFCRs in the R4 region. It is of great interest to us that TFCRs regulate gene expression at various stages of embryonic development through dynamic changes in their location accessibility.

**Fig. 4:**
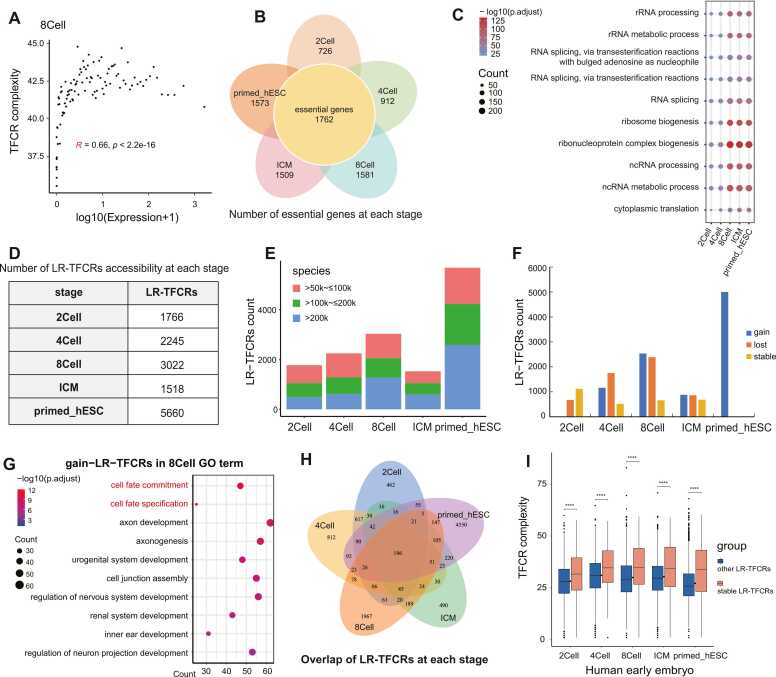
p-TFCRs & LR-TFCRs. **A.**Scatterplot of p-TFCR complexity versus associated gene expression levels at 8Cell stage shows a strong correlation between them. R represents the magnitude of correlation. **B.**Number of essential genes contained in the associated genes of p-TFCRs in each stage. **C.**Gene ontology enrichment analysis of essential genes in each stage. **D.**Number of LR-TFCRs accessibility in each stage. **E.**The number of LR-TFCRs accessibility in each stage. Stacked colour bars indicate the number of tfcrs located at different distances. **F.**The number of gained and lost LR-TFCRs in each stage compared to the consecutive stages. Gained TFCRs were compared to the previous stage. Lost TFCRs were compared to the next stage. **G.**Gene ontology enrichment analysis of gained LR-TFCRs in 8Cell. **H.**Veen diagram shows the overlap of LR-TFCRs in each stage. **I.**Distribution of TFCR complexity between stable LR-TFCRs and the other LR-TFCRs at various stages of embryonic development displays the higher complexity of stable-LR-TFCRs, ****p ≤ 0.0001, ***p ≤ 0.001, **p ≤ 0.01, *p ≤ 0.05, ns p > 0.05.

First of all, those R1 region TFCRs, which we call proximal TFCRs (p-TFCRs), also showed a strong correlation between TC and gene expression level at early embryonic developmental stages, and at the same time, showed a clear trend of increasing correlation at 8Cell and later stages (Fig. 4A, Fig. S4a). We explored the ratio of essential gene-related TFCRs in R1-R4, and the results showed that essential gene-related TFCRs were mainly distributed in the R1 region with the development of embryos (Fig. S4b). The dynamic changes in the location of accessible TFCRs that we identified in early human embryonic development are consistent with critical life incidents such as ZGA during human embryonic development to ensure the continuation of the normal developmental process of the embryo. We hypothesize that p-TFCRs act similarly to the promoter region of genes and can regulate gene expression. Therefore, we calculated the proportion of promoter sequence elements in p-TFCRs. Here we download a list of core promoter elements from the CORE database (https://www.juven-gershonlab.org/resources/core-2023/). We co-locate sequences of these elements with p-TFCRs, and the result shows that the proportion of elements in p-TFCRs is similar to that in promoter region (Fig. S4c). We counted the number of essential genes contained in the genes regulated by p-TFCRs at each stage. And we found that the 8Cell, ICM, and primed-hESC stages respectively contained 1581, 1509, and 1573 essential genes, more than 85 % of essential genes[39] (Fig. 4B). Moreover, we found that these essential genes were mainly enriched in ribonucleoprotein complex biogenesis, ncRNA metabolic process, and rRNA processing to regulate transcription (Fig. 4C). In summary, we believe that p-TFCRs contain promoter regions and have the function of regulating gene expression. All of them can bind transcription factors and other regulatory proteins, thereby affecting the transcriptional activity of downstream genes. The results show that most of the essential genes in early embryonic development are regulated by p-TFCRs.

We defined TFCRs in more distant R4 regions (>50k distance from the gene) as long-range TFCRs (LR-TFCRs, Supplementary Table 2). First, we counted the number of accessible LR-TFCRs at each stage of embryonic development (Fig. 4D) and divided them again by distance (Fig. 4E). We found that LR-TFCRs can reach over 200k. Our previous study also showed that LR-TFCRs still exist in mature tissues, albeit in limited numbers. Next, we analyzed the specific dynamic changes of LR-TFCRs accessibility at each stage, identifying the LR-TFCRs gained, lost, and kept stable at each stage (Fig. 4F). The changes in LR-TFCRs accessibility showed the most dramatic changes at the 8Cell stage. GO enrichment analysis of 8Cell gained LR-TFCRs-associated genes was mainly enriched in cell fate specification and cell fate commitment (Fig. 4G), which may be related to human ZGA in 8Cell stage.

We used deeptools to overlap the positions of TFCRs at various stages of early embryonic development. A stable LR-TFCR is defined as one that overlaps more than 1 bp in each stage. The result reveals the intriguing presence of 196 conserved accessible LR-TFCRs across all developmental stages (Fig. 4H, Supplementary Table 3). We hypothesize that during the early stages of embryogenesis, the positioning of accessible TFCRs undergoes dynamic shifts, with the chromatin initially opening at random, then selectively closing, and ultimately stabilizing in regions that are functionally significant. This hypothesis prompted us to delve deeper into the characteristics of these LR-TFCRs. In our examination, we observed that stable LR-TFCRs exhibit a higher transcriptional complexity (TC) compared to their counterparts (Fig. 4I). Moreover, these stable regions are broader in width, yet paradoxically, they tend to have a lower expression level (Fig. S4d). This result shows that stable LR-TFCRs may not be directly involved in immediate gene regulation but could be poised for later developmental roles. Further investigation led us to identify the genes associated with stable LR-TFCRs, which intriguingly, demonstrate a consistent presence accessibility across all stages (Fig. S4e). This consistency implies that the regulatory influence of stable LR-TFCRs may be situated at a considerable distance from the genes themselves, often in so-called 'gene deserts.' Gene Ontology enrichment analysis of these associated genes (Fig. S4f), indicates a significant enrichment in functions related to the chromatin-mediated maintenance of transcription, positive regulation of gene expression, and the intricate interplay of epigenetic and circadian regulation of gene expression. Given the relatively low expression levels of genes associated with stable LR-TFCRs, we speculate that these regulatory elements may not exert their influence by directly modulating gene expression. Instead, they could shape the three-dimensional architecture of chromatin, thereby indirectly influence gene activity. However, how LR-TFCRs play a regulatory role during early human embryonic development needs to be explored in depth.

### 1.4 Result 4 LR-TFCRs may mediate the establishment of three-dimensional chromatin through phase separation

**Fig. 5:**
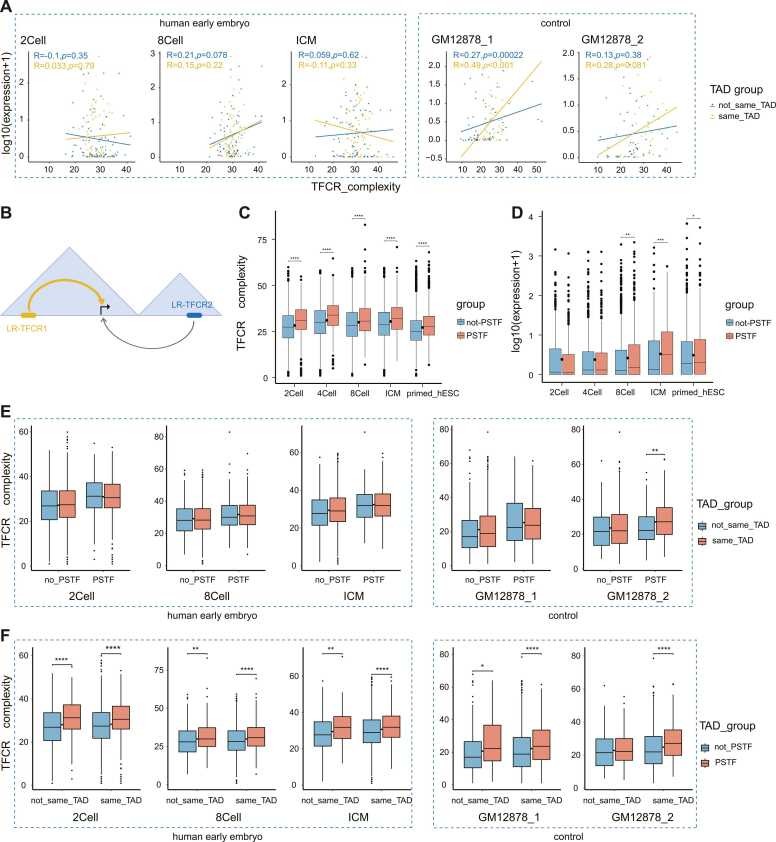
LR-TFCRs may mediate the establishment of 3D chromatin through phase separation effects. **A.**Scatterplot of the LR-TFCRs complexity at each sample correlated with gene expression levels in not_same_TAD vs. same_TAD groups, the cor.test() function was used to calculate the correlation coefficient, and the parameter of method was set as "spearman". R stands for correlation coefficient between two data. p represents the significant level of correlation. **B.**Schematic illustration of LR-TFCRs transcriptional regulation models. **C.**Distribution of the LR-TFCRs complexity in the not_PSTF vs. PSTF group shows a higher TC in PSTF group, ****p ≤ 0.0001, ***p ≤ 0.001, **p ≤ 0.01, *p ≤ 0.05. **D.**Distribution of the LR-TFCRs related gene expression levels in the not_PSTF vs. PSTF group displays a higher expression level in PSTF group, ****p ≤ 0.0001, ***p ≤ 0.001, **p ≤ 0.01, *p ≤ 0.05. **E.**Distribution of LR-TFCR complexity of PSTF vs. LR-TFCRs of not_PSTF in the not_same_TAD vs. same_TAD group, ****p ≤ 0.0001, ***p ≤ 0.001, **p ≤ 0.01, *p ≤ 0.05. **F.**Distribution of LR-TFCR complexity of not_same_TAD versus LR-TFCRs of same_TAD in the not_PSTF versus PSTF group, ****p ≤ 0.0001, ***p ≤ 0.001, **p ≤ 0.01, *p ≤ 0.05.

Distal enhancers mostly function by forming certain spatial proximity with the help of three-dimensional genomic changes [40]. Previous studies [14], [15], [16]have shown that the structure of three-dimensional chromatin undergoes reprogramming during early embryonic development and builds up slowly with developmental stages. The regulatory role of LR-TFCRs may be related to three-dimensional chromatin reprogramming with developmental progress. To this end, we co-localized 2cell, 8Cell, and ICM (TADs are derived from the blastocyst) LR-TFCRs with the corresponding TADs[16], and then grouped LR-TFCRs with their associated gene positions according to whether they existed within the same TAD or not. The scatterplot of TFCRs complexity versus gene expression level (log10(Expression+1)) was plotted by dividing the genes into 100 groups according to the order of expression. In the early embryo, whether or not a TFCR is within the same TAD, it has little effect on the correlation between complexity and expression (Fig. 5A). GM12878 is a widely used human lymphoblastic cell line, they were added as a mature sample to explore the dynamic changes of TFCRs from early embryonic development to maturity. Following the study reporting the gradual establishment of three-dimensional chromatin structure, we added GM12878 and found that the correlation between LR-TFCRs complexity and the expression of its associated genes within the same TAD was also stronger than within the non-same TAD (Fig. 5A). We hypothesize that the reason for this phenomenon is that TADs are gradually established during early embryonic development. The above results show that TFCRs distributed within the same TAD as the associated genes have a slightly stronger correlation. In summary, we conjecture that TFCRs distributed within the same TAD as the associated genes have a somewhat stronger regulatory role than the not-same TAD as the related genes (Fig. 5B).

To date, we still do not know how transcription factors cluster to coordinate gene expression during early embryonic development. Recent studies have shown that transcriptional bursts are regulated by dynamic transcription factor clustering on enhancers[41]. The regulatory connection between TF clustering and burst induction is essentially highly regulated through intrinsically disordered regions (IDRs). These regions are thought to promote dynamic molecular clustering through multivalent protein-protein interactions, which we also commonly refer to as phase separation interactions.

To explore the LR-TFCRs regulatory mechanism, we downloaded the files of phase separators from LLPSDB v2.0 [42] (http://bio-comp.org.cn/llpsdbv2) to screen out the transcription factors related to human phase separations (Supplementary Table 4). And we identified bindable TFs on LR-TFCRs and labeled phase-separation-associated transcription factors (PSTFs). We categorized LR-TFCRs according to the presence or absence of PSTFs, PSTF (LR-TFCRs containing PSTFs), and not-PSTF (LR-TFCRs not containing PSTFs). Then we explored the complexity and distribution of genes associated with LR-TFCRs in these two groups (Fig. 5C-D), and the results show that TC and gene expression levels of TFCRs in the PSTF group are higher than the not-PSTF group. Consistent with the result observed with LR-TFCRs, TFCRs in the R1-R3 region also showed higher levels of TC in the PSTF group (Fig. S5a-c). We found that TC and associated gene expression level of TFCRs in the PSTF group was significantly higher than that in the not-PSTF group. Next, we explored whether the number and percentage of PSTFs also had an effect (Fig. S6a-d), and the result showed that the number and percentage of PSTFs had less effect on LR-TFCRs-associated genes. The most important factor was the presence of PSTFs. We conjecture that LR-TFCRs achieves their transcriptional regulatory effects on genes through phase-separation. We also analyzed the effect of PSTFs on LR-TFCRs-associated genes in the GM12878 cell line (Fig. S6e-j). The result also showed that the increased number and percentage of PSTFs did not influence gene regulation in GM12878. In summary, we hypothesize that binding of LR-TFCRs with PSTF binding sites to PSTF induces a phase-separation effect recruiting more TFs to bind to these LR-TFCRs, which exerts the corresponding regulatory effects.

We also conducted an overall study of LR-TFCRs at different embryonic developmental stages and in the two GM12878 groups, and found that the complexity of LR-TFCRs present within the same TAD in the PSTF group in GM12878 was significantly higher than that in the no_TFCRs group (Fig. 5E). The complexity of LR-TFCRs in the PSTF group was significantly higher regardless of whether they existed within the same TAD or not (Fig. 5F). The results showed that differences were exhibited in the presence of PSTF in all samples, whereas TADs showed differences only in mature systems. We conjecture that this is a difference since the three-dimensional chromatin structure is established gradually during early embryonic development. Taken together, we conjecture that LR-TFCRs may play a regulatory role in the process of promoting the establishment of chromatin by PSTFs-mediated phase separation. We conjecture that LR-TFCRs containing phase-separation-associated transcription factors may mediate the establishment of three-dimensional chromatin through phase-separation effects. The establishment of a three-dimensional chromatin structure can help regulate gene expression at the spatial level.

## 2 Discussion

The coordinated roles of transcription factors and CREs during early embryonic reprogramming are unclear. The gene regulation we are concerned with is much more complex, with multiple transcription factors capable of binding to DNA sequences in an orderly fashion and functioning properly to accomplish regulatory actions on genes at specific stages of development. Additionally, multiple elements play a key role in gene regulation. Not only CREs and multiple transcription factors, but also histone modification, DNA methylation, and changes in chromatin structure all work together in a coordinated manner to enable the embryo to complete normal development. The regulation of gene expression during each stage of early embryonic development is cell-specific. Upon the formation of the zygote, cellular chromatin accessibility, histone modifications, and three-dimensional chromatin structure undergo reprogramming, with gene regulation dynamically evolving.

Therefore, we identified human early embryonic TFCRs and calculated the complexity of TFCRs to investigate the dynamics of gene regulation during early embryonic development. The complexity of TFCRs represents the number of TFBSs in the region as well as their ability to bind to transcription factors. By calculating TFCRs and their complexity, we can more precisely identify regulatory elements from open chromatin data and perform quantitative evaluation of regulatory elements.

**Fig. 6:**
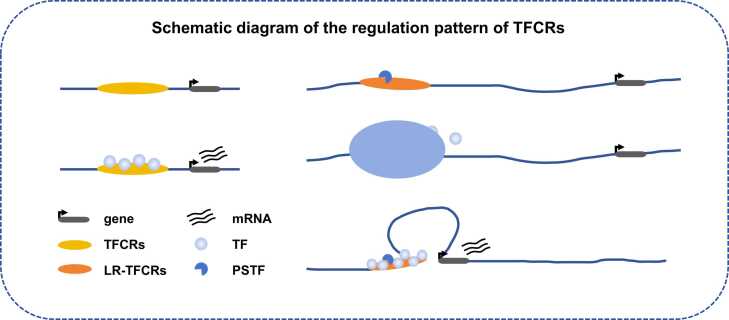
A model illustrating the multiple modes of action of TFCRs during development in the human early embryo. Proximal TFCRs bind to TFs and exert direct regulatory effects on genes. LR-TFCRs with phase-separated protein binding sites bind to PSTFs, aggregate other related TFs with the help of phase-separation, promote changes in chromatin structure, and pull in the spatial distance between LR-TFCRs and associated genes, thus realisingregulatory effects.

Interestingly, we found a strong correlation between TFCR complexity and width of accessible TFCRs versus gene expression from 8Cell onwards, which is consistent with the dramatic changes in gene expression that occur in human embryos from the 8Cell stage onwards. And exploring TFCRs according to their distance and location from the gene transcription start site (TSS), we found that as early embryonic development progresses, the accessibility of TFCRs dynamically changes with development and gradually stabilizes. TFCRs in different genomic regions may serve different roles. As a regulatory region co-localized with the promoter region, we suppose that p-TFCRs, being co-localized with the promoter region, serve a similar role. We found that more than 85 % of the genes associated with p-TFCRs are essential genes. In other words, essential genes are regulated by proximal TFCRs to maintain normal function. Proximal TFCRs exercise transcriptional regulatory functions by acting directly on genes (Fig. 6).

The phenomenon of phase separation is widespread in cells, where thousands of protein or nucleic acid molecules exercising their specific functions aggregate into distinct droplets within the cell and participate in a variety of important biological processes such as transcriptional regulation, stress, protein quality control, DNA replication, and so on, without interfering with one another within the cell [43], [44]. Several studies have shown that phase separation is one of the important regulators of the three-dimensional structure of chromatin. Transcription factors can promote the reconstruction of chromatin three-dimensional structure through phase separation, and then control cell fate transitions. In contrast, the phase separation of abnormal proteins in the nucleus leads to misfolded chromatin. This misfolding can promote disease development [45]. Phase separation of OCT4, a major transcription factor for somatic cell reprogramming, can promote cell fate transitions by affecting the chromosomal three-dimensional structure and regulating key genes controlling cell identity [46]. In 2022, it was demonstrated that CTCF possesses a novel function to mediate long-distance chromatin interactions and a new function was proposed for CTCF to regulate very long-distance chromatin interactions in a new model [47]. Previous review [45] has summarized three models of phase separation and chromatin three-dimensional structure: (1) phase separation regulates the expression of cell type-specific genes by regulating the formation of chromatin structure, thus promoting cell fate transition; (2) specific chromatin three-dimensional structure regulates the expression of cell type-specific genes by promoting the formation of phase separation; (3) chromatin three-dimensional structure and phase separation each regulate the expression of genes.

Here, we also defined and identified LR-TFCRs and found that the accessibility of LR-TFCRs may gradually stabilize which refers to their enhanced accessibility and/or functional stability within the cell, which may be indicative of their regulatory activity. We presume that they play a regulatory role in gene transcription during the PSTFs mediated phase separation effect and the continuous establishment of chromatin (Fig. 6). We conjecture that phase separation is one of the important mechanisms regulating the formation of three-dimensional chromatin structures. We presume that at the early stages of embryonic development, the regulation of gene expression levels by TFCRs exhibits: transcription factor enrichment, transcription surge, and saturation state. The binding of PSTFs to LR-TFCRs induces aggregation of transcription factors. We conjecture that the spatial state between LR-TFCR and related genes changes under phase separation, thus exerting its regulatory role. Therefore, we presume that LR-TFCRs regulate the progressive formation of three-dimensional chromatin structures in early embryos through phase separation to regulate genes.

In conclusion, we have identified the regulatory patterns of TFCRs during early human embryonic development through chromatin accessibility analysis. This will promote efforts to establish a gene regulation model for the early human embryo and provide assistance in further resolving gene regulatory relationships in the early human embryo.

## 3 Methods

### 3.1 Dataset collection

Human early embryonic ATAC-seq peaks and gene expression data were obtained from the GSE101571[8] which were downloaded from the GEO database (https://www.ncbi.nlm.nih.gov/geo/). ATAC-seq peaks and gene expression of GM12878_1, Ovarian, and Uterine were obtained from the GSE188405[48]. ATAC-seq peaks and gene expression of GM12878_2, ovary, and uterus were downloaded from ENCODE (https://www.encodeproject.org/). GM12878 is a widely used human lymphoblastic cell line, often used as a model system for studying human gene expression and epigenetic properties. Uterine and ovarian samples represent mature tissue of the reproductive system, but they differ significantly in function and development from early embryos. These samples provide a baseline for gene expression and regulation in mature tissues and help us understand regulatory changes during early embryonic development. ATAC-seq peaks and gene expression data of 23 cancer samples were obtained from the TCGA database (https://portal.gdc.cancer.gov/repository). These samples were considered mature cancer tissue samples. ATAC-seq peaks of GM12878_2, ovary, and uterus reference genome we converted from GRCH38/hg38 to GRCH37/hg19 as reference. All data analysis used GRCH37/hg19 (http://www.repeatmasker.org/) as a reference. Phase-separated proteins were downloaded from the PhaSeqDB database [49].

### 3.2 Identification of TFCRs

First, we applied FIMO [33] to identify TFBSs. The position-specific weight matrices (PWMs) of transcription factors were downloaded from the CIS-BP database [34]. The genomic sequence of the chromatin accessibility region in the hg19 genome was used as input to FIMO to scan for motif instances. Then, an established [29] method was used to identify TFCRs by performing Gaussian kernel density estimation of the entire genome (centered at 300 bp). Each peak in the density profile was considered to be a TFCR. To determine the complexity of TFCRs, we determined the Gaussian kernalised distance between each TFCR and each peak that contributed at least 0.1 to its intensity. The complexity of each TFCR was determined by the number and proximity of the contributing TFBSs. We calculated the complexity of TFCRs based on the TF family information from CIS-BP, combined with motif instances. This means that if multiple motifs are combined at the same locus, we only calculate the complexity of the different TF families they belong to as TFCRs. TFCRs Complexity(TC) scores are determined based on the number of TFBS predicted within TFCRs, reflecting the diversity and abundance of transcription factors within a given region.

### 3.3 Identification of TFCRs-associated genes

To unify the criteria of regulatory action, we assume that genes are regulated by TFCRs which is closest to the gene TSS. We obtained human genome position information from the R package (TxDb.Hsapiens.UCSC.hg19.knownGene). Circulate each gene and TFCR in all samples, compare the distance between the gene transcription start site (TSS) and each TFCR, and filter the TFCR closest to the TSS of each gene, which serves as the associated gene of this TFCR. If more than one TFCR had the same distance from the gene TSS, all were retained.

### 3.4 The unit base complexity (cw)

We define the ratio of the complexity of the TFCRs to the width of the TFCRs as the unit base complexity of the TFCRs (cw).

### 3.5 Classification of TFCRs

We centered on each TFCR compared the distance between the TFCR and the transcription start site (TSS) of the gene, and filtered the nearest gene to the TFCR for tagging. If more than one gene had the same distance to the TFCR, all were retained, thus obtaining a matrix of TFCR-gene distances. Then we explored the density distribution of the TFCR-gene distance matrix for five samples of early embryos and selected the distances of 2kbp,27kbp, and 50kbp to categorize the TFCRs.

### 3.6 Phase separation-related transcription factors

We downloaded the related files of phase separators from LLPSDB v2.0 [42] (http://bio-comp.org.cn/llpsdbv2) to screen out the transcription factors related to phase separations in Homo sapiens.

### 3.7 Statistical analysis

In this study, gene Expression level (FPKM, PKM) was uniformly normalized, and Expression+ 1 (log10) was calculated as a unified standard to measure gene expression level.

y = log10(x + 0.03) was determined by lm() function from R-package to fit the regression model. rsquare() function was used to evaluate the fit model.

In this study, the cor.test() function was used to calculate the correlation coefficient, and the parameter of the method was set as "Spearman". R stands for correlation coefficient between two data. p represents the significant level of correlation. When p < 0.05 was specified, the correlation and difference were considered to be statistically significant (*, p < 0.05; **, p < 0.01; ***, p < 0.001; ****, p < 0.0001).

In this study, the t-test was used to compare the difference of pairwise data in the difference analysis of boxplot. When p < 0.05 was specified, the difference was considered statistically significant (*, p < 0.05; **, p < 0.01; ***, p < 0.001; ****, p < 0.0001). When ns is specified, the difference is not considered statistically significant.

### 3.8 Gene ontology (GO) enrichment analysis

We used the R software package "clusterProfiler" for Gene Ontology (GO) enrichment analysis. The parameter Settings for analysis include keyType = " ENTREZID", OrgDb = org.Hs.eg.db, pvalueCutoff = 1, qvalueCutoff = 1, ont= "BP", readable =T. We considered GO terms with pvalue < 0.05 as significantly enriched categories.

## Authors’ contributions

Mengge Tian was involved in data analysis, experimental procedures, and drafting of the manuscript. Xiaohan Tang participated in data analysis and funding acquisition. Zhangyi Ouyang and Yaru Li participated in data analysis and revised the manuscript. Xuemei Bai participated in data analysis. Bijia Chen revised the figures. Shutong Yue and Pengzhen Hu participated in the data analysis. Xiaochen Bo, Chao Ren, Hebing Chen, and Meisong Lu oversaw the project and guided the research direction. Xiaohan Tang Hebing Chen and Meisong Lu secured the funding. All authors reviewed and approved the final version of the manuscript.

## Funding

This work was supported by the 10.13039/501100001809National Natural Science Foundation of China (http://www.nsfc.gov.cn; No. 62173338 to H.Chen; No.62203463 to C.Ren; No.82001520 to X.Tang), Beijing Nova Program of Science and Technology (20220484198 to H.Chen), WU JIEPING MEDICAL FOUNDATION (https://www.wjpmf.org.cn; No. 320.6750.2021–04-27 to M.Lu) and the First Affiliated Hospital of Harbin Medical University Research and Innovation Fund (https://www.54dr.org.cn; No.2021M22 to X.Tang).

## CRediT authorship contribution statement

**Bijia Chen:** Formal analysis. **Yaru Li:** Methodology, Formal analysis. **Xuemei Bai:** Data curation. **Xiaohan Tang:** Funding acquisition, Data curation. **Zhangyi Ouyang:** Writing – review & editing, Writing – original draft, Methodology, Formal analysis, Data curation, Conceptualization. **Hebing Chen:** Writing – review & editing, Supervision, Project administration, Methodology, Investigation, Funding acquisition. **Mengge Tian:** Writing – review & editing, Writing – original draft, Resources, Project administration, Methodology, Investigation, Formal analysis, Data curation, Conceptualization. **Meisong Lu:** Supervision, Funding acquisition. **Xiaochen Bo:** Supervision, Funding acquisition. **Chao Ren:** Writing – review & editing, Supervision, Project administration, Methodology. **Shutong Yue:** Methodology. **Pengzhen Hu:** Methodology.

## Ethics approval and consent to participate

Not applicable.

## Consent for publication

Not applicable.

## Declaration of Competing Interest

All authors declare that they have no conflict of interest.

## Data Availability

The code for TFCR identification and all the results of identified TFCRs are available on github (https://github.com/MenggeTian-77/TFCRs).
