# Supplementary material for: Long-range transcription factor binding sites clustered regions may mediate transcriptional regulation through phase-separation interactions in early human embryo
Source: Comput Struct Biotechnol J. 2024 Sep 26;23:3514–26. doi: 10.1016/j.csbj.2024.09.017 (PMC11492133; doi:10.1016/j.csbj.2024.09.017)
Supplement: Supplementary file 5 — Supplementary material [file mmc5.pdf]

Fig. S1

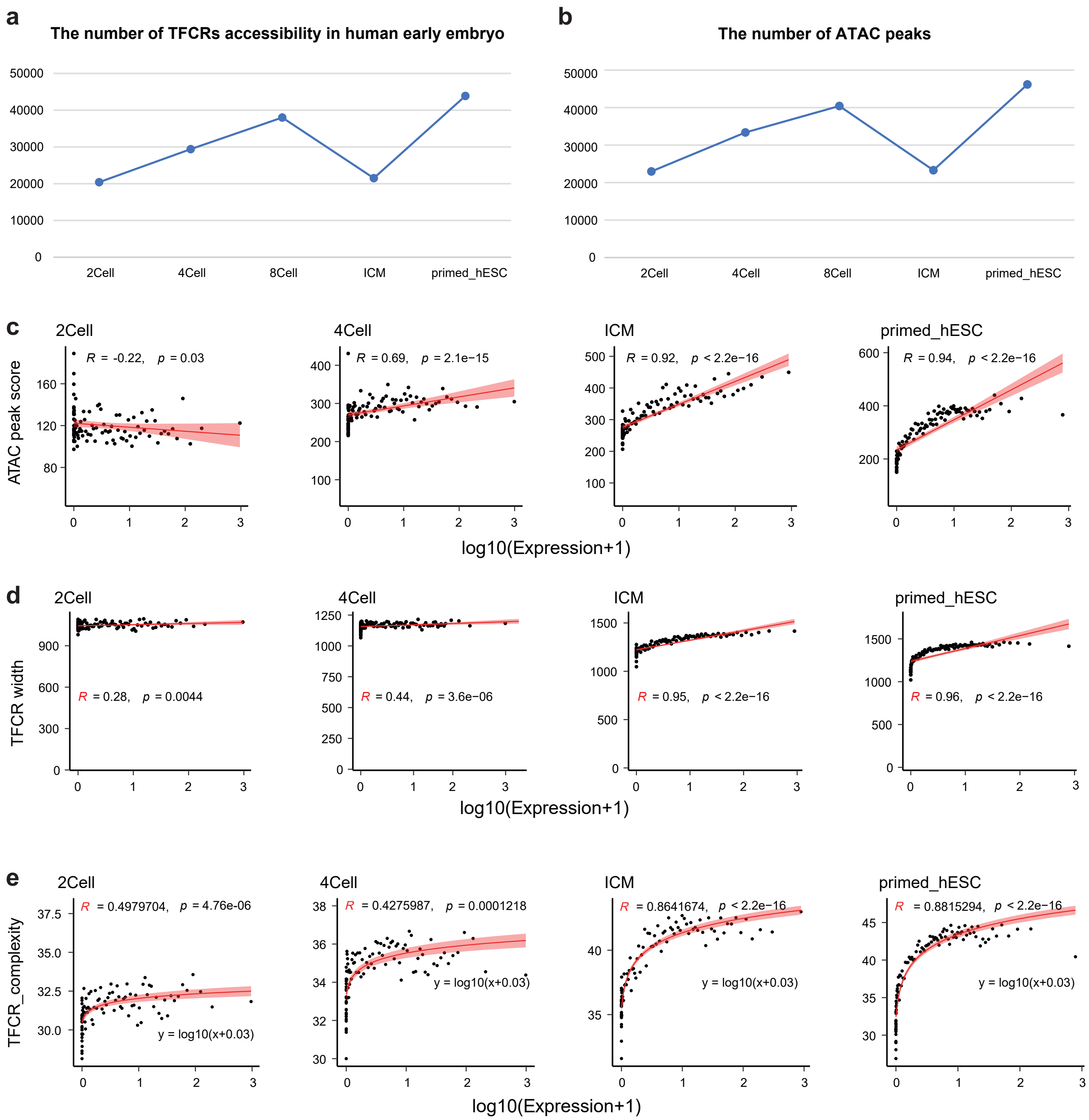

Fig.S1 | Correlation of TFCRs characteristics with gene expression levels. a. The number of TFCRs accessibility in human early embryo. **b.** The number of ATAC peaks in human early embryo. **c.**Scatterplot of ATAC peak score versus gene expression levels. The red part: fitting of ATAC peak score with gene expression, R represents the correlation magnitude. **d.**Scatterplot of TFCR width versus gene expression level. Red section: fitting of TFCR width to gene expression, R represents the magnitude of correlation. **e.**Scatterplot of TFCR complexity versus gene expression levels. The red part: fitting of TFCR complexity with gene expression, R represents the correlation magnitude.

Fig. S2

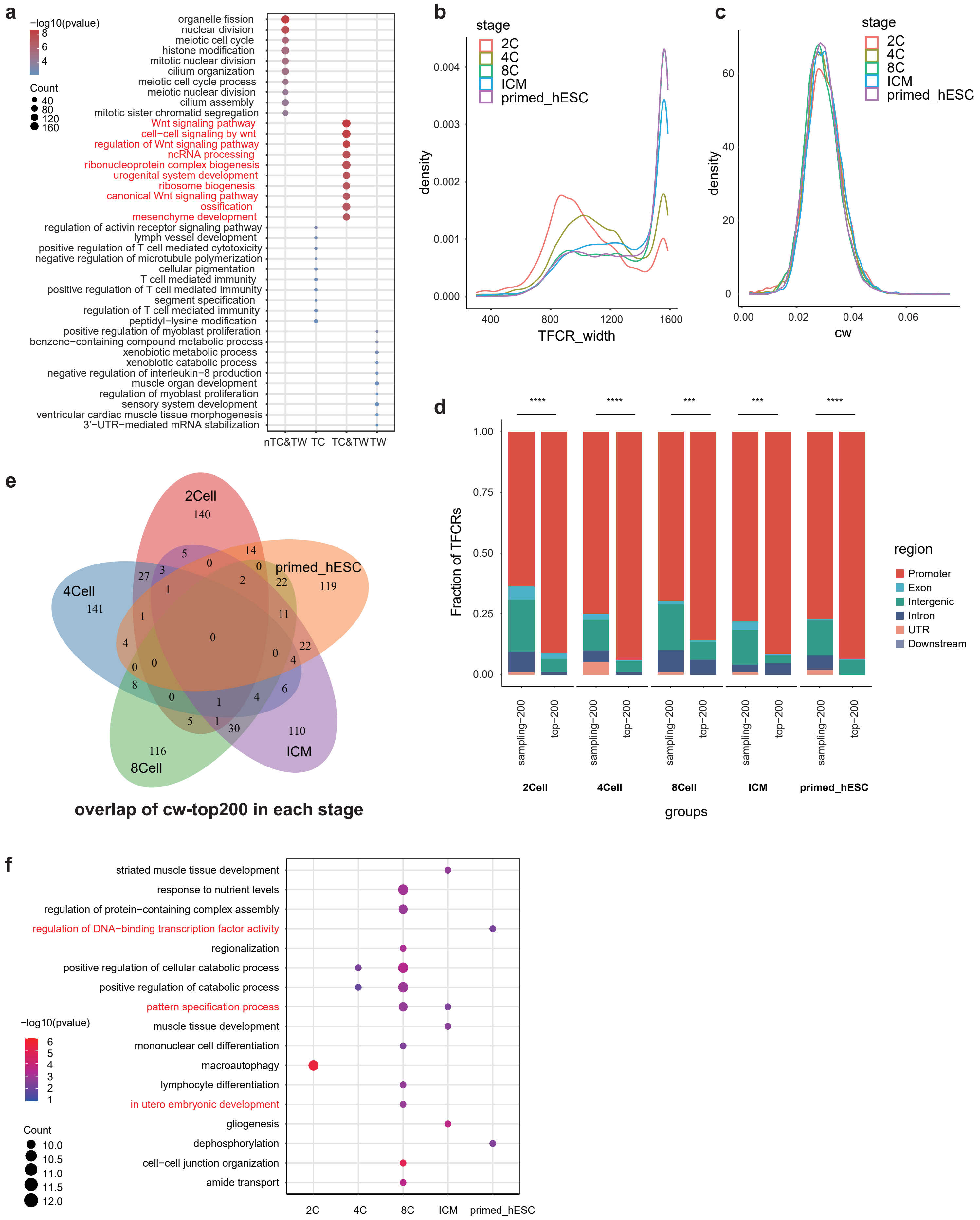

Fig.S2 | TFCR complexity and TFCR width. a. Gene ontology enrichment analysis of relevant genes in nTC&TW, TC, TC&TW, and TW group. **b.**Density distribution plot of the TFCR width. **c.**Density distribution plot of the cw of TFCRs. **d.**The genomic distribution of TFCRs identified in each stage of sampling-200 group vs. top-200 group.Use the prop.test() function to perform a proportional hypothesis test. ****$p \le 0.0001$,***$p \le 0.001$, **$p \le 0.01$, *$p \le 0.05$. **e.**Overlapping vennplot of genes associated with 200 (top-200) TFCRs with high cw in descending order of cw value. **f.**Gene ontology enrichment analysis of genes associated with top-200 TFCRs at various times in the early embryo (count>10).

Fig. S3

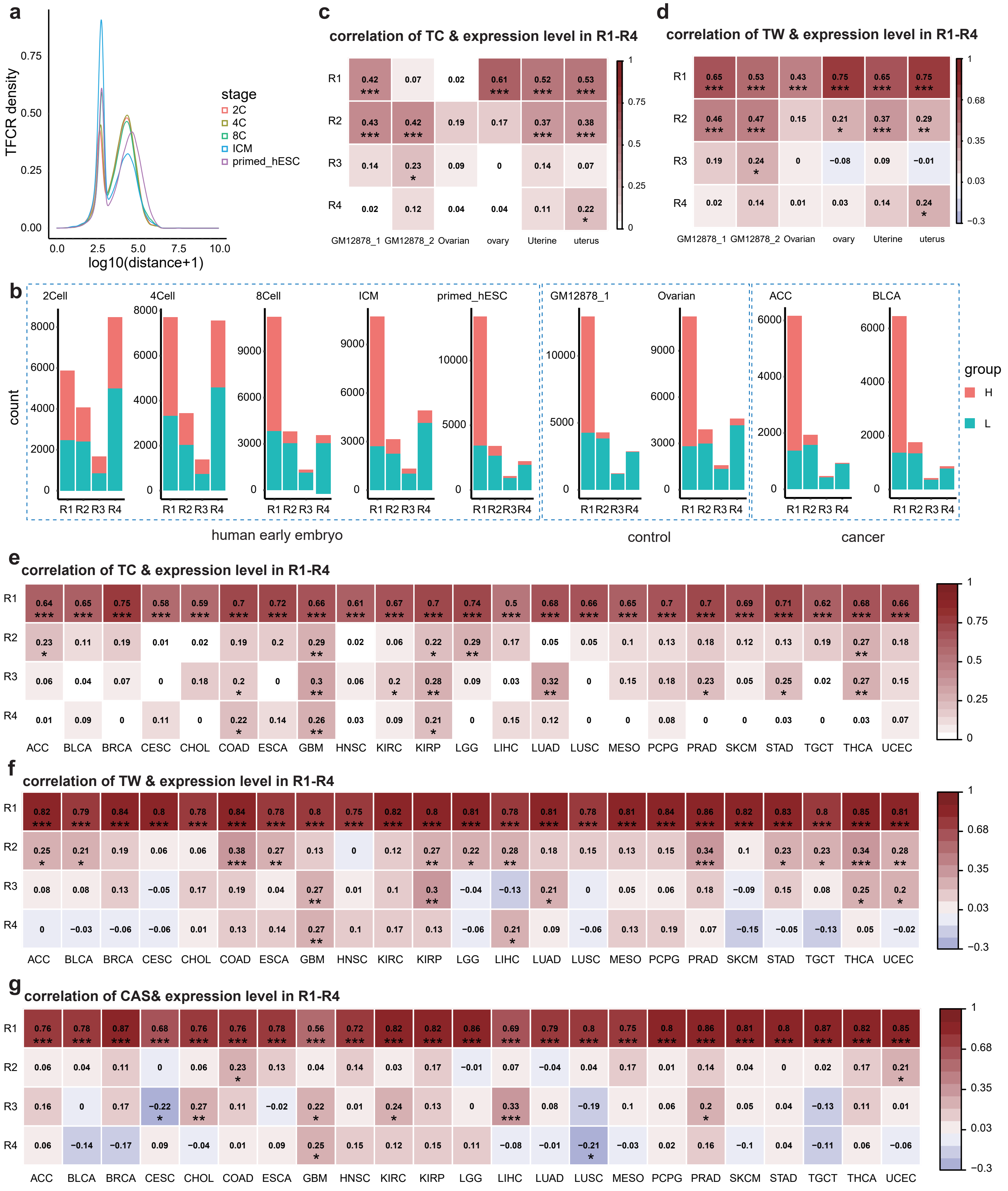

Fig.S3 | Correlation between the expression and characterization of TFCR in R1-R4. a. Density distribution plot of the distance between TFCRs and genes associated with TFCRs. The horizontal axis is the normalized distance. **b.**Number of TFCRs-associated genes per region in each sample in the high-expression group (H group) versus the low-expression group (L group). **c.**Heatmap showing the correlation between the expression of TFCR-associated genes in each region and TFCR complexity in normal samples. **d.**Heatmap showing the correlation between the expression of TFCR-associated genes in each region and TFCR width in normal samples, ***p ≤ 0.001, **p ≤ 0.01, *p ≤ 0.05. **e.**Heatmap showing the correlation between the expression of TFCR-associated genes in each region and TFCR complexity in tumor samples. **f.**Heatmap showing the correlation between the expression of TFCR-associated genes in each region and TFCR width in tumor samples, ***p ≤ 0.001, **p ≤ 0.01, *p ≤ 0.05. **g.**Heatmap showing the correlation between the expression of TFCR-associated genes in each region and TFCR CAS in tumor samples, ***p ≤ 0.001, **p ≤ 0.01, *p ≤ 0.05.

Fig. S4

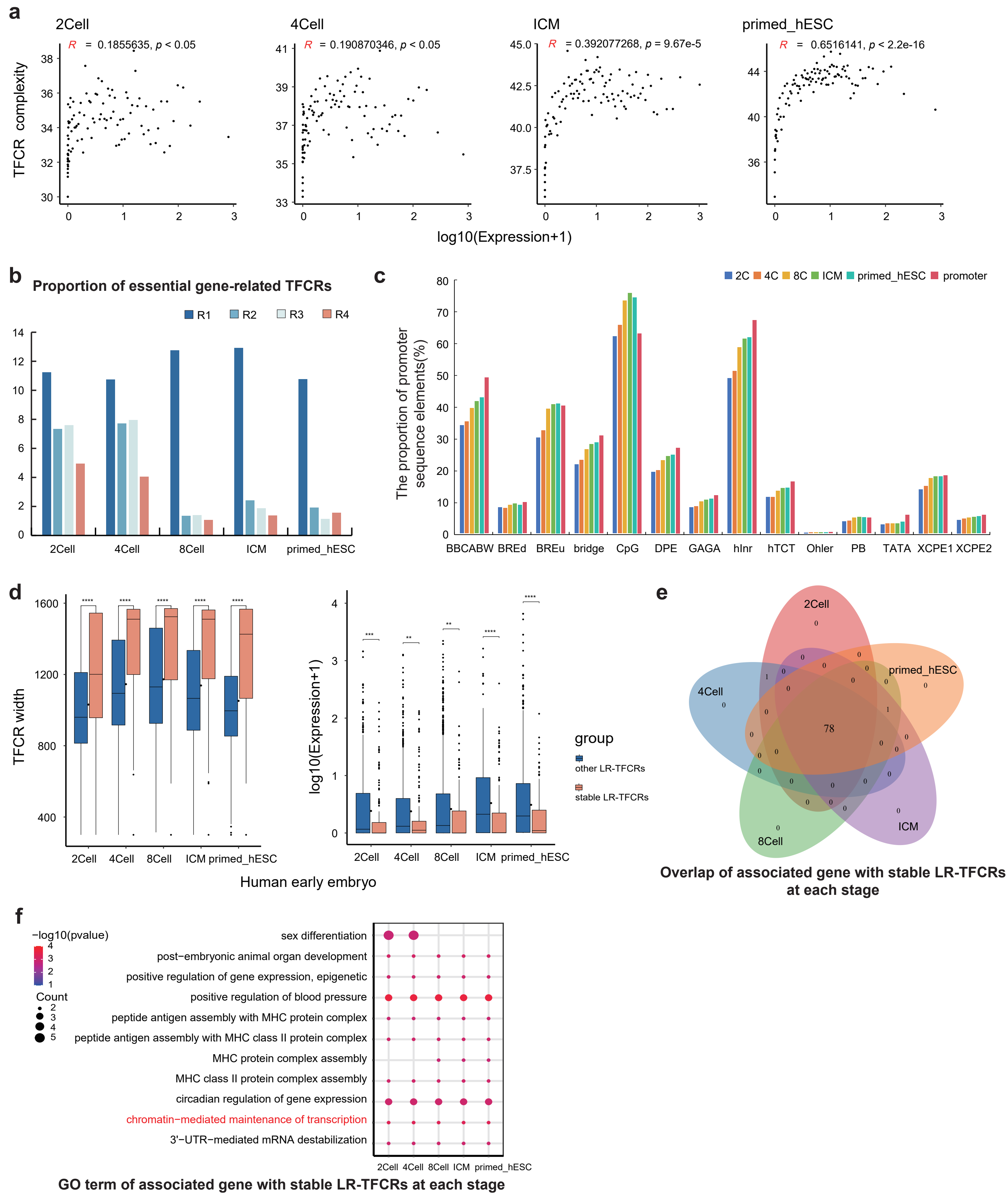

Fig S4 | Stable LR-TFCRs. a. Scatterplot of p-TFCR complexity versus expression level of p-TFCR-associated genes. Red section: fitting of p-TFCR complexity versus gene expression, R represents the magnitude of the correlation. **b.**The proportion of essential gene-related TFCRs in R1-R4. **c.**The proportion of promoter sequence elements in p-TFCRs and promoter (2k bp). **d.**Distribution of TFCR width and expression levels of TFCRs-associated genes between stable LR-TFCRs and the other LR-TFCRs at various stages of embryonic development, ****p ≤ 0.0001, ***p ≤ 0.001, **p ≤ 0.01, *p ≤ 0.05, ns p > 0.05. **e.**Overlapping vennplot of genes associated with stable LR-TFCRs at each stage. **f.**Gene ontology enrichment analysis of genes associated with the stable LR-TFCRs at various stages.

Fig. S5

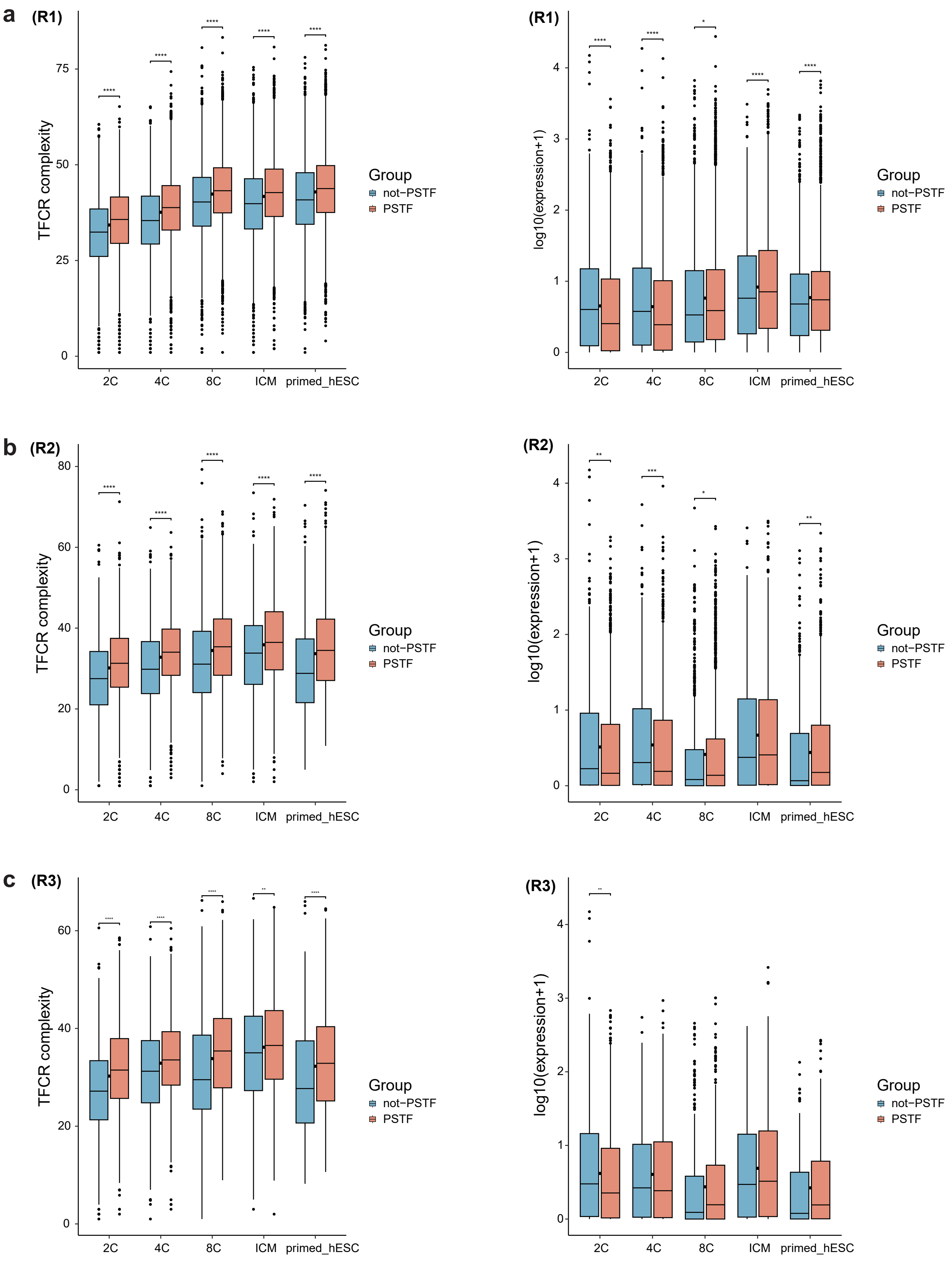

Fig S5 | Distribution of the R1-R3 region TFCRs complexity and gene expression level in the not_PSTF vs. PSTF group.

Fig. S6

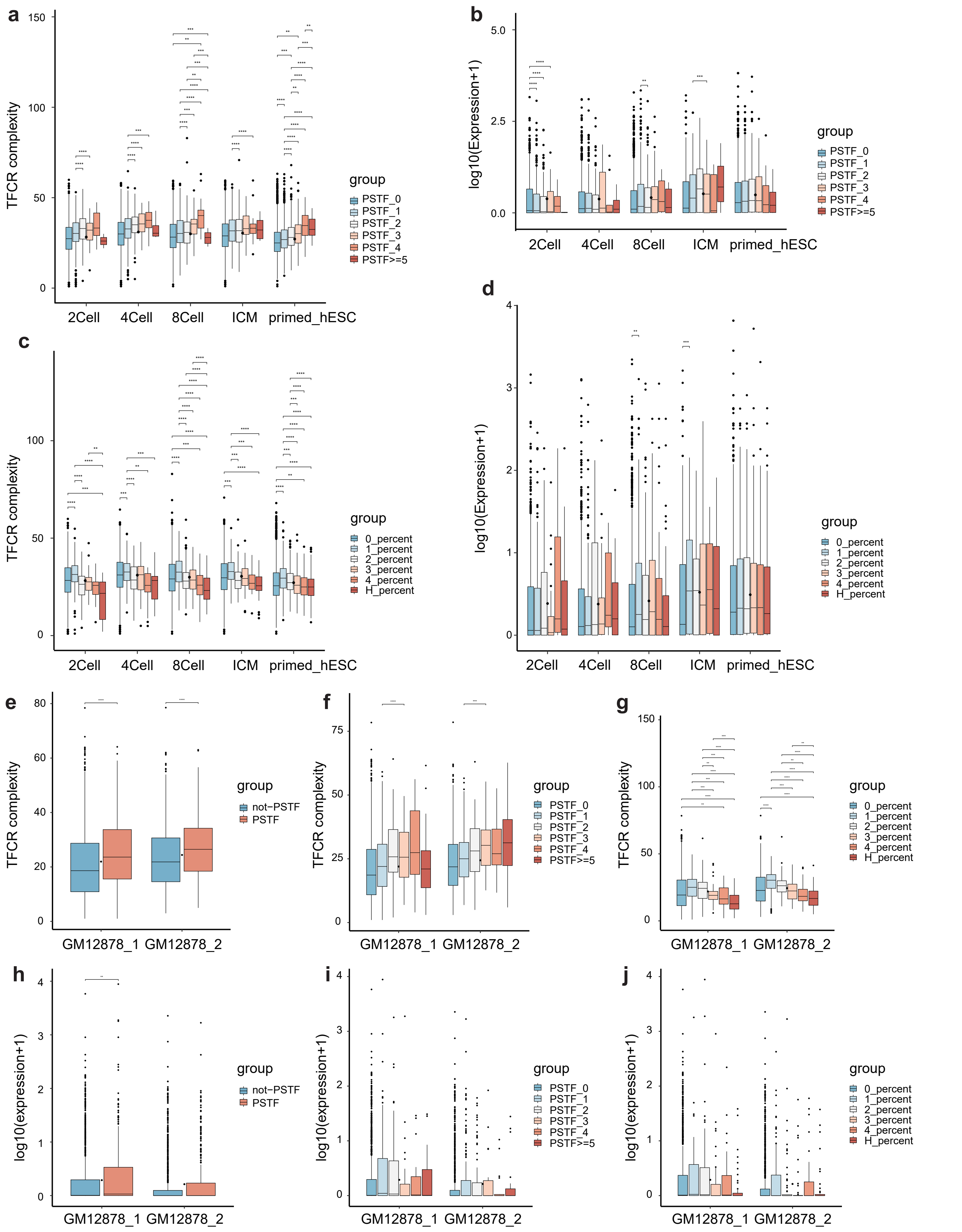
a
b
c
d
e
f
g
h
i
j
TFCR complexity
log10(Expression+1)
log10(expression+1)
2Cell
4Cell
8Cell
ICM
primed_hESC
GM12878_1
GM12878_2
group
PSTF_0
PSTF_1
PSTF_2
PSTF_3
PSTF_4
PSTF>=5
0_percent
1_percent
2_percent
3_percent
4_percent
H_percent
not−PSTF
PSTF

Fig S6 | LR-TFCRs may mediate the establishment of 3D chromatin through phase separation effects in different samples. a. Boxplots of LR-TFCR complexity distribution in different numbers of PSTF groups. **b.**Boxplots of LR-TFCRs-associated gene expression levels in different numbers of PSTF groups. **c.**Boxplots of LR-TFCR complexity distribution in different proportions of PSTF groups. **d.**Boxplots of LR-TFCRs-associated gene expression levels in different proportions of PSTF groups. **e.**Boxplot of the distribution of LR-TFCR complexity in GM12878 in the not-PSTF vs. PSTF group shows a higher TC in PSTF group. **f.**Boxplot of LR-TFCR complexity in GM12878 distribution in different numbers of PSTF groups. **g.**Boxplot of LR-TFCR complexity in GM12878 distribution in different proportions of PSTF groups. **h.**Boxplot of LR-TFCRs-associated gene expression levels in GM12878 distributed in not-PSTF vs. PSTF groups. **i.**Distribution boxplot of LR-TFCRs-associated gene expression levels in GM12878 across different numbers of PSTF groups. **j.**Distribution boxplot of LR-TFCRs-associated gene expression levels in GM12878 across different proportions of PSTF groups. (t-test was used to compare the difference of pairwise data in the difference analysis of boxplot, ****$p \leq 0.0001$, ***$p \leq 0.001$, **$p \leq 0.01$, *$p \leq 0.05$)
